# Immediate breast reconstruction using latissimus dorsi muscular flap: A retrospective study of Chinese patients with breast cancer

**DOI:** 10.1097/MD.0000000000026175

**Published:** 2021-06-18

**Authors:** Qiuming Liu, Weifeng Li, Xiaobo Wu, Liang Xu, Pinghua Hu, Yali Cao

**Affiliations:** Department of Breast Surgery, Breast Cancer Institute, The Third Hospital of Nanchang, Nanchang, China.

**Keywords:** breast cancer, immediate breast reconstruction, implants, latissimus dorsi myocutaneous flap, mastectomy

## Abstract

Reconstruction of breast defects of patients who underwent mastectomy can be challenging. This study was designed to review a series of 43 breast cancer patients who underwent immediate breast reconstruction (IBR) using the latissimus dorsi myocutaneous flap with/without implants. The demographic characteristics, clinical application feasibility, and the satisfaction rates of the patients were retrospectively collected and evaluated.

A total of 43 breast cancer patients who underwent mastectomy between August 2015 and February 2020 were included in the retrospective study. The included patients were subjected to IBR using latissimus dorsi muscular flap (LDMF) with/without implants. The clinical application feasibility and the satisfaction rates of the patients were evaluated.

Among these patients, 35 patients underwent nipple-sparing mastectomy and 8 patients underwent skin-sparing mastectomy. Twenty-nine patients underwent IBR using LDMF with implants, and 14 patients underwent IBR using LDMF without implants. Among these patients, 2 patients had partial LDMF necrosis and atrophy, and showed significant shrink of the reconstructed breast. One patient developed seromas, and seromas were improved by active dressing change and sucking out the fluid via the skin using a syringe. Two patients had local skin flap necrosis on the chest, 1 patient had preserved areola and local necrosis of the nipple, and this was healed after dressing change. Based on the Harris method, 27, 9, 5, and 2 cases were evaluated as “excellent,” “good,” “fair,” and “poor,” respectively.

In the present study, the reconstructed breast has natural shape, good symmetry, and hidden postoperative scars. The aesthetic effect is relatively good, and the use of LDMF may represent an acceptable and valid option for IBR. The success of this procedure depends on the design of the incision, the skill and proficiency of the operation, as well as the correct treatment after surgery.

## 1 Introduction

Breast cancer is one of the most common human malignancies in women.^[1]^ Among the breast cancer patients, about 30% patients received mastectomy.^[2]^ These patients after mastectomy often complained about changes in body shape, impaired posture, decrease in the concept of femininity and self-confidence, which may result in depression and anxiety in these patients.^[3,4]^ Thus, breast reconstruction is a proper strategy to maintain the functional and emotional features in these patients.

Breast reconstruction refers to the formation of a breast bulge resembling the previous breast shape after mastectomy. The application of this procedure has been increasing in recent years.^[5]^ Autologous tissues have been commonly used in the breast reconstruction in patients after breast radical surgery.^[6–8]^ Among these autologous tissues, the latissimus dorsi muscular flap (LDMF) remains an acceptable and valid option, due to its outstanding aesthetic outcomes and well-known anatomy.^[9]^ Although LDMF is an acceptable option, several concerns such as complications and donor-site disadvantages have been raised.^[10,11]^ Though breast reconstruction using LDMF has been reported in several studies, little information has been available regarding clinical outcomes following immediate breast reconstruction (IBR) in the Chinese population. Moreover, there are no detailed investigations that specifically address the possible risks and complications. This study was designed to review a series of 43 breast cancer patients who underwent IBR using the LDMF with/without implants. The demographic characteristics, clinical application feasibility, and the satisfaction rates of the patients were retrospectively collected and evaluated.

## 2 Patients and methods

### 2.1 Patients

A total of 43 patients who underwent skin-sparing mastectomy between August 2015 and February 2020 were included in the retrospective study. The included patients were subjected to IBR using l LDMF with/without implants. The clinical characteristics of the included patients were summarized in Table 1. The inclusion criteria were as follows: age < 60 years old; newly diagnosed breast cancer according to the eighth edition of AJCC (American Cancer Council) TNM staging standard in 2017; the clinical staging is 0 to IIb; the patients had good cardiopulmonary function; no contraindications to surgery. The exclusion criteria were as follows: age ≥ 60 years old; breast-conserving surgery can be performed; stage IIIa and above; skin invasion; computed tomography or magnetic resonance imaging examination excludes tumor invasion of adjacent organs, metastasis elsewhere; poor cardiopulmonary function; unable to tolerate the surgery. The study was approved by the Ethics Committee of The Third Hospital of Nanchang, and all the patients signed the written informed consent.

**Table 1:** Clinical characteristics of the patients.

Clinical parameters	Number of patients
Tumor grade	
1	14
2	16
3	13
Tumor size	
<3 cm	20
≥3 cm	23
Lymph node metastasis	
Yes	9
No	34
Nipple-sparing mastectomies	
Yes	35
No	8
Implants	
Yes	29
No	14
Breast cancer types	
Luminal A	9
Luminal B	19
HER2 positive	8
Basal-like	7

### 2.2 Surgical techniques

Before surgery, doppler ultrasound and mammography were performed to determine the tumor location, size, and distance from the nipple–areola area, and assess whether the nipple–areola complex can be preserved. The condition of the subscapular blood vessels and the thickness of the latissimus dorsi were also examined by doppler ultrasound. Preoperative assessment of the systemic condition was performed to rule out the existence of hidden infections. Preoperative measurement of breast volume was used to determine size and model of the implants to ensure that the reconstructed breast and the healthy side are symmetrical and beautiful. During the surgery, the patient was placed in a lateral position with the upper arm located on an armrest in an abducted position. The skin island was drawn into a horizontal position and the width of the paddle was measured according to skin previously resected and to produce an easy closure (4–10 cm, depending on resection). The peripheral limits of the muscle were also determined and marked on the skin surface. The inferior and superior flap extension was subjectively estimated to match the volume of glandular tissue removed. The dissection proceeded in the muscular plane in the caudal direction until the iliac crest and cranially until the scapular bone. Close dissection of the thoracodorsal vessels was not necessary, but the thoracodorsal nerve was identified and divided to prevent potential postoperative involuntary muscle contraction. The vascular branch to the serratus anterior muscle was divided, if it limited flap rotation. The flap was passed under the axillary tunnel to the breast defect region and the patient turned to the supine position to perform flap shaping. In this position, the division of the humeral attachment of the muscle was performed only when necessary to obtain adequate excursion. Two drains were inserted (dorsal and breast), and the dorsal closure was performed in 3 layers. The color of the flap after the operation was monitored. Patients with implants were routinely treated with the first-generation cephalosporin antibiotics for 28 hours. The volume and color of the drainage fluid were monitored every day.

### 2.3 Patient follow-up

After IBR surgery was completed, all patients were followed-up and examined from 3 to 52 months. Cosmetic results were evaluated by using Harris method. An “excellent” rating means that the treated breast was nearly identical to the untreated breast; a “good” rating means that the treated breast was slightly different from the untreated breast; a “fair” rating means that the treated breast was not seriously distorted but clearly different from the untreated breast; while a “poor” rating means that treated breast was seriously distorted.

## 3 Results

### 3.1 Clinical characteristics of patients

The clinical characteristics of recruited patients were shown in Table 1. The age range of the cases was 28 to 59 years old (mean: 43.9 years old). For the tumor grade, 14, 16, and 13 patients were classified into grade 1, 2, 3, respectively. For the tumor size, 20 patients had tumor size <3 cm and 23 patients had tumor size ≥3 cm. Nine patients had lymph node metastasis, and the rest ones had no lymph node metastasis. For the breast cancer types, the number of patients with luminal A, luminal B, HER2 positive, or basal-like was 9, 19, 8, and 7, respectively.

### 3.2 Clinical outcomes after IBR in the recruited patients

**Figure 1:**
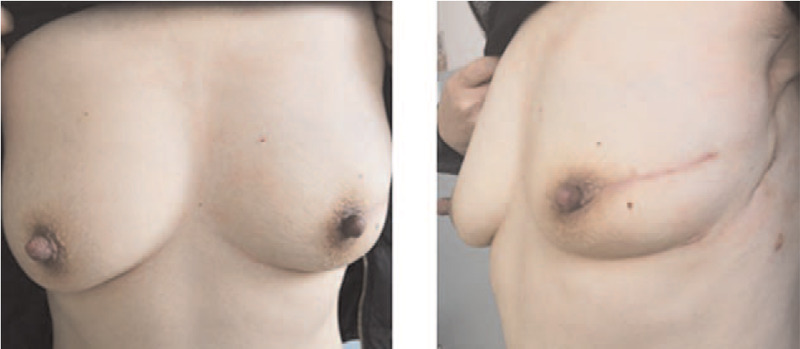
The front and lateral views of the patient who underwent NSM using the LDMF with implants after 6 mo. LDMF, latissimus dorsi myocutaneous flap; NSM, nipple-sparing mastectomy.

**Figure 2:**
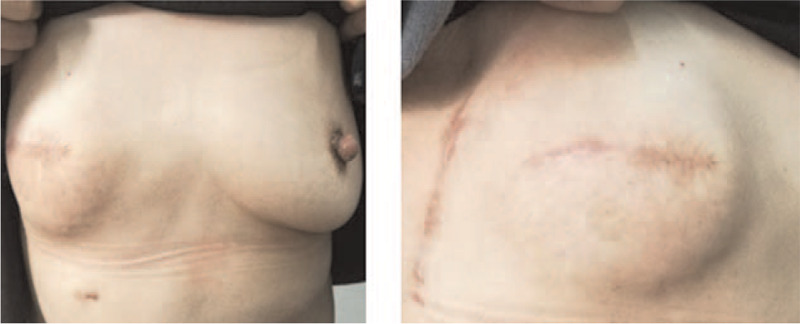
The front and lateral views of patient who underwent SSM using the LDMF with implants after 6 mo. LDMF, latissimus dorsi myocutaneous flap; SSM, skin-sparing mastectomy.

Thirty-five patients received nipple-sparing mastectomy; 8 patients received skin-sparing mastectomy. The lateral and front view of the patient received nipple-sparing mastectomy after 6 months were illustrated in Figure 1; while the lateral and front views of the patient received skin-sparing mastectomy were shown in Figure 2.

**Figure 3:**
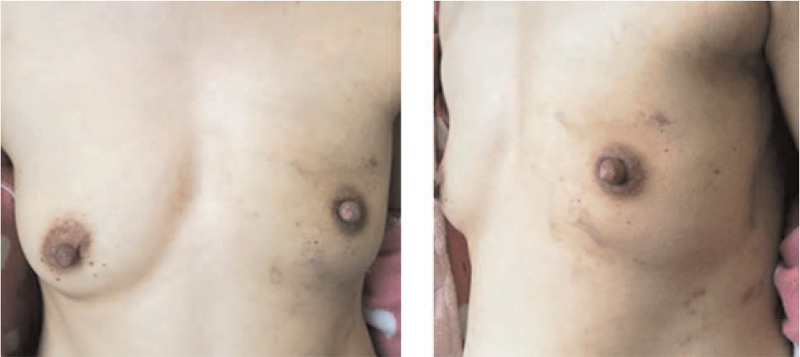
The front and lateral views of the patient who underwent NSM using the LDMF without implants after 6 mo. LDMF, latissimus dorsi myocutaneous flap; NSM, nipple-sparing mastectomy.

Twenty-nine patients underwent IBR using LDMF with implants (Fig. 1) and 14 patients underwent IBR using LDMF without implants (Fig. 3). Among these patients, 2 patients had partial LDMF necrosis and atrophy, and showed significant shrink of the reconstructed breast. One patient developed seromas, and seromas were improved by active dressing change and sucking out the fluid via the skin using a syringe. Two patients had local skin flap necrosis on the chest; 1 patient had preserved areola and local necrosis of the nipple, and this was healed after dressing change.

### 3.3 Clinical outcomes of the follow-up

The mean follow-up period was 19.0 months (range 3–52 months). Two patients underwent implant removal due to postoperative infection and capsular contracture. The Harris score of the patients was summarized in Table 2. Based on the Harris method, 27, 9, 5, and 2 cases were evaluated as “excellent,” “good,” “fair,” and “poor,” respectively. In the patients received implants, 19, 5, 3, and 2 cases were evaluated as “excellent,” “good,” “fair,” and “poor,” respectively; while in the patients without receiving implants, 8, 4, 2, and 0 cases were evaluated as “excellent,” “good,” “fair,” and “poor,” respectively. All the patients had no tumor recurrence or tumor metastasis.

**Table 2:** Harris score from the included patients.

Harris score	Number of patients	
	With implants	Without implants
Excellent	19	8
Good	5	4
Fair	3	2
Poor	2	0

## 4 Discussion

The first choice of treatment for early breast cancer is breast-conserving surgery. However, some patients are not qualified for breast-conserving surgery, and some patients concerned the risk of recurrence.^[12,13]^ Breast reconstruction brings new options for these patients, which retains the shape of the breast and significantly improves the quality of patients’ life.^[14,15]^ Breast reconstruction with only implants is limited to patients whose reconstructed breasts are small in size and can retain more soft tissue as a covering.^[16]^ However, the reconstructed breasts are usually unnatural and not symmetrical with the contralateral breast. Moreover, the rate of infection and capsular contracture are relatively high.^[16]^ IBR using LDMF with/without implants has the advantages of simple operation, safety, concealed back scar, and can fill subclavian defects and form breast axillary folds, which is especially suitable for patients who have not given birth and wish to have children.

Breast reconstruction should be considered aspects of the safety of cancer treatment and cosmetic effects. In one aspect, IBR is aimed to achieve the ideal cosmetic and functional effects and improved the quality of patients’ life; on the other hand, IBR should not interfere with breast cancer treatment, not affect the immediate detection and retreatment of tumor recurrence.^[17–20]^ Several types of patients were considered suitable for this operation:

(a) patients with stage I and II breast cancer;
(b) middle-aged and young patients, who have a strong need for breast reconstruction;
(c) those without breast-conserving indications requiring modified radical mastectomy.^[17–20]^

Distant metastasis is an absolute contraindication to breast reconstruction.^[21–23]^ Patients with stage III or IV breast cancer and with tumor invading the skin and muscle layer have a poor prognosis, and these patients should not undergo reconstruction surgery.^[21–23]^ It is best to choose early breast cancer patients who are unlikely to need radiotherapy after surgery.^[24]^ If the patient's ipsilateral breast has received radiotherapy or subsequent radiotherapy is required, it may affect the cosmetic effect of reconstructed breasts. In addition, these patients more likely to have complications such as infection, protrusion deformation, capsular contracture, implants exposure, etc. In addition, this operation is suitable for modified radical mastectomy. The LDMF has a small amount of tissue and can be combined with the implant to reconstruct a satisfactory breast shape. If the implant cannot be filled (such as after radical operation), it can only be used breast reconstruction with skin flaps such as the rectus abdominis muscle with more tissue. In addition, if the contralateral breast is excessively droopy, the contralateral breast needs to be reshaped, otherwise it will cause breast asymmetry. There are 29 patients who received IBR using LDMF with implants in this study. In this study, a total of 83.7% patients were satisfied with the aesthetic results, and our results were comparable to previous studies using deep inferior epigastric perforators flaps and implants for breast reconstruction (more than 80% patients were very happy or very happy with the aesthetic results).^[25,26]^ Clinically, if there is diffused benign lesions, precancerous lesions, family history, and high-risk factors for breast cancer that are difficult to clean in the healthy side breast. This type of preventive resection can be recommended to prevent future malignant transformation of the healthy breast while making the appearance of the breasts more beautiful and symmetrical.

There are several concerns, which should be considered for this surgery. All patients are confirmed to be breast cancer by frozen pathological examination before or during the operation; patients with nipple-preserving areola should be at least 3 cm from the nipple at the edge of the tumor and no nipple discharge. During the operation, the posterior nipple tissue should be histologically examined to that no cancerous tissue remains. A total of 29 patients in this group underwent this operation. Because the nipple and areola were preserved, the postoperative cosmetic effect was significantly better than the breast reconstruction effect after conventional modified radical mastectomy. During the operation, attention should be paid to protecting the thoracic nerve and blood vessels to prevent postoperative chest muscle atrophy.

There are several limitations in our study. Firstly, this study was a single-center retrospective study, and the included sample size was relatively small. Thus, the inherent bias would inevitably occur. Secondly, the small number of clinical adverse events might have a certain impact on the research results. Thirdly, the occurrence of clinical events was obtained by telephone or outpatient follow-up, which might inevitably lead to reporting bias.

## 5 Conclusions

The use of LDMF combined with implants for breast reconstruction is a safe and feasible way to improve the breast cosmetic effect after breast cancer surgery. The success of this procedure depends on the design of the incision, the skill and proficiency of the operation, as well as the correct treatment after surgery. In the present study, the reconstructed breast has natural shape, good symmetry, and hidden postoperative scars. The aesthetic effect is relatively good, and the use of LDMF may represent an acceptable and valid option for IBR.

## Author contributions

**Conceptualization:** Qiuming Liu, Yali Cao.

**Data curation:** Qiuming Liu, Xiaobo Wu, Yali Cao.

**Formal analysis:** Weifeng Li, Xiaobo Wu.

**Funding acquisition:** Yali Cao.

**Investigation:** Qiuming Liu, Weifeng Li, Yali Cao.

**Methodology:** Liang Xu.

**Writing – original draft:** Yali Cao.

**Writing – review & editing:** Pinghua Hu.
